# CuO-NiO-Embedded Guar Gum Hydrogel as an Efficient Catalyst for Dyes Removal

**DOI:** 10.3390/polym17111577

**Published:** 2025-06-05

**Authors:** Nujud Maslamani

**Affiliations:** 1 Department of Physical Sciences, Chemistry Division, College of Science, Jazan University, P.O. Box. 114, Jazan 45142, Saudi Arabia; nmaslamani@jazanu.edu.sa; 2 Engineering and Technology Research Center, Jazan University, P.O. Box 114, Jazan 82817, Saudi Arabia

**Keywords:** guar gum, catalytic reduction, dyes, organic pollutants, metal oxide, hydrogel

## Abstract

In this work, Guar gum and copper oxide-nickel oxide (GG-CuO-NiO) hydrogel were produced with the help of formaldehyde solution to display an efficient catalytic performance toward the catalytic degradation of selected dyes (Methylene Blue (MB), Methyl Orange (MO), and Eosin Yellow (EY)) in the presence of NaBH_4_. The morphological and structural properties of the prepared hydrogel were thoroughly analyzed using SEM, EDX, XRD, and FT-IR techniques. According to the results, the GG-CuO-NiO hydrogel was able to reduce MB by 95% in one minute, 90.0% in four minutes, and 80.0% in 10 min for MO and EY, respectively. The catalytic efficiency of the hydrogel for MB was studied by adjusting its concentrations, varying reducing agent concentrations, and altering the amount of gel used. Using the recyclability method, which involved testing the GG-CuO-NiO hydrogel multiple times for the reduction of MB, the stability, reusability, and loss of catalytic activity of the hydrogel were examined. As a result, the designed GG-CuO-NiO hydrogel was stable for up to four times toward the reduction of MB. Lastly, the efficiency of the GG-CuO-NiO hydrogel was evaluated for MB removal in real samples and displayed exceptional reduction capabilities.

## 1. Introduction

The issue of water pollution has escalated over time. It poses a threat to both human wellbeing and the natural environment’s health. The rise in development and activity levels has led to an increase in the release of wastewater into water bodies, leading to a decline in water quality and the contamination of aquatic environments. Various industrial sectors, such as textile and pharmaceutical industries, often release dyes and inorganic pollutants into wastewater, which possess chemical properties that require careful consideration. The presence of these substances in water sources can disrupt the balance of ecosystems by contaminating food chains and accumulating in living organisms [1,2,3].

The textile, paper, and pharmaceutical industries widely use synthetic dyes. However, a significant portion of these dyes [4], often more than 50%, remains unbound to textile fibers and is released into wastewater [4]. Among the commonly used dyes are Methyl Orange (MO), Methylene Blue (MB), and Eosin Y (EY). While MO is a representative azo dye, MB and EY belong to the thiazine and xanthene dye classes, respectively. Despite their structural differences, these dyes share similar physicochemical characteristics: they are highly water-soluble, chemically stable, and toxic to aquatic organisms. Their presence in water bodies poses serious environmental and public health concerns. Therefore, the development of efficient and versatile removal methods capable of addressing multiple dye classes has become a critical environmental priority [4,5,6,7,8].

Catalysis plays a role in achieving effective results in water treatment processes as researchers focus their efforts on developing catalysts using cleaner and more efficient methods for the easier separation and reuse of catalysts. Of the various catalytic materials, metal oxides have been shown to be very effective catalysts because of their high surface area, easily tuned electronic properties, and excellent stability [9,10]. Moreover, metal oxides are usually inexpensive, recyclable, and environmentally friendly, and are thus well suited for sustainable catalytic processes. Studies have explored copper oxide and nickel oxide nanoparticles as nanocatalysts due to their properties such as high surface to volume ratio and reactivity. However, the practical application of metal oxides is hindered by several weaknesses. A major problem with metal oxides is their tendency to aggregate, thus decreasing the active surface area and catalytic activity. In addition, the difficulty handling powdered metal oxides due to their small size can lead to their loss during filtration cannot; they are not easily recovered and may be dangerous to inhale or disperse in the environment. As a result, low cycling stability and the requirement of a complicated separation process increase the operating cost and hinder their large-scale use [11,12,13,14,15,16,17,18,19].

Incorporating metal oxides into polymer matrices offers an effective approach to address the challenges faced in metal oxide catalysts. The embedding of metal oxide nanoparticles into a polymer matrix helps minimize problems related to aggregation, difficult handling, and catalyst loss during separation. CMC-based nanocomposites demonstrate outstanding catalytic properties when they are combined with metal oxide nanoparticles for organic dye degradation applications. The combination of chitosan with metal oxides creates effective adsorption systems for heavy metals and catalytic pollutant reduction because of their strong chelating properties and reactive amine groups. The gel-forming properties of alginate enable the immobilization of nanoparticles, which enhances their reusability and improves their dispersion [20,21].

Another promising biopolymer is guar gum (GG), derived from guar plant Cyamopsis tetragonolobus (Cluster bean), which is widely used as a thickener and stabilizer in a variety of food products, including baked goods, soups, sauces, and dairy products. In a 1:2 ratio, galactose and mannose units make up guar galactomannan. Guar gum has a high molecular weight, ranging from 0.1 to 2.8 million, which accounts for its high viscosity. It is composed of a linear backbone of β-1,6-linked mannose units with ἂ-1,6-linked galactose units randomly attached as side chains. Food, pharmaceuticals, cosmetics, textiles, and oil recovery are just a few of the industries that employ it because of its excellent viscosity and low cost. Nonetheless, the variety of uses for modified guar galactomannan make it valuable. The modification of guar gum, especially by incorporating metal oxides into a three-dimensional hydrogel matrix, minimizes problems such as aggregation, poor dispersion, and powder handling. This integration not only enhances catalytic efficiency but also enhances recyclability and reusability, which makes it a promising strategy for sustainable water treatment [22,23,24]. Polymeric hydrogels, with their hydrophilic networks, provide strong support for nanoparticles, acting as efficient catalysts and adsorbents. The three-dimensional structure, along with a high surface area, facilitates the accommodation of nanoparticles, creating abundant catalytic sites that enhance reaction rates and efficiency [25,26,27,28,29,30].

The primary goal of this research is to develop a 3D network hydrogel by combining GG with CuO-NiO nanocomposite materials, effectively enhancing its strength and properties. The GG-CuO-NiO nanocomposite hydrogel was examined as a catalyst for the selected dyes MO, MB, and EY, using SEM analysis along with EDS, FT-IR and XRD techniques utilized for the analysis of the resulting hydrogel. The prepared hydrogel is cost-effective and highly efficient in eliminating dyes.

## 2. Experimental

### 2.1. Material

Guar gum (GG) powder was obtained from Sigma Aldrich. All reagents were analytical and spectral purity grade. Metal salts of Nickel(II) sulfate (NiSO_4_·6H_2_O, (99.999%)), Copper(II) sulfate (CuSO_4_·5H_2_O ≥ 98.0%), and organic dyes ((Methylene Blue (MB), Methyl Orange (MO), Eosin Yellow (EY)), sodium borohydride (NaBH_4_), urea, formaldehyde, and NaOH were obtained from Sigma-Aldrich. Deionized water was employed throughout all synthesis and preparation steps.

### 2.2. Synthesis of CuO-NiO

The CuO-NiO nanocomposite was created using a coprecipitation method [31,32] where (0.1 M) CuSO_4_ and (0.1 M) NiSO_4_ were mixed in a 1:1 ratio. NaOH was added drop by drop until the pH reached 11. After 4 h of preparation at 80 °C with continuous stirring to allow for precipitation formation, the product was filtered and then cleaned multiple times with distilled water before being left to dry on the laboratory bench for 24 h at a temperature of 25 °C. Additionally, the samples underwent calcination at 500 °C, for a duration of 4 h.

### 2.3. Synthesis of GG-CuO-NiO Hydrogel

**Figure 1:**
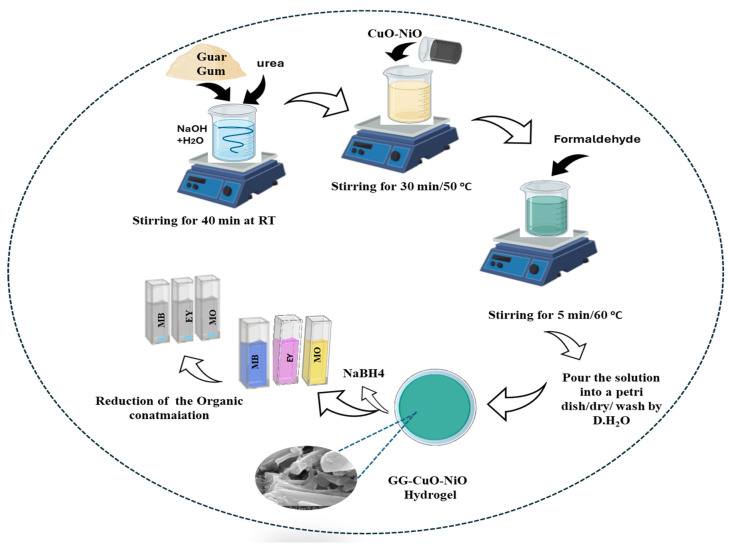
Schematic representation of the preparation of GG-CuO-NiO hydrogel and 29 the catalytic study.

The GG solution was prepared by dissolving 1.5 g of GG powder with 3.5 g of NaOH and 6 g of urea in 50 mL of distilled water and stirring for 40 min. Then, 60 mg of CuO-NiO nanocomposite was added to the GG solution with complete stirring for 30 min. Finally, 5 mL of formaldehyde was added to the mixture with stirring for 5 min under 60 °C, and then the mixtures were poured into a Petri dish. The resulting GG-CuO-NiO hydrogel was gently rinsed three times with distilled water to remove the excess reactants, as shown in Figure 1.

### 2.4. Instrumental Analysis

A field emission scanning electron microscope (FE-SEM of JEOL, JSM-7600F, Japan) was used to examine the samples for the morphological investigation. For FE-SEM analysis, CuO-NiO nanocomposite, GG hydrogel, and GG-CuO-NiO hydrogel were individually mounted on stubs using carbon tape as an adhesive and sputter-coated with platinum for 15 s. Elemental analysis of GG-CuO-NiO hydrogel was analyzed by an energy dispersive spectrometer (EDS). Thermo Scientific equipment was used to measure the samples’ X-ray diffraction (XRD) patterns in order to determine the crystal structure, and from 5° to 50° (2θ), diffraction patterns were found. UV–vis spectra, measured using a Thermo Scientific TM Evolution TM 350 UV–vis spectrophotometer, were used to record the catalytic reduction studies. An FT-IR spectrometer (NicoletTM iS50 FT-IR Spectrometer-Thermo Fisher Scientific) was used to record the Fourier transform infrared (FT-IR) spectra of the samples in the range of 400–4000 cm^−1^.

### 2.5. Catalytic Reduction

To test the catalytic activity of synthesized GG-CuO-NiO hydrogel, it was sliced into small pieces with a knife. Initially, 2.5 mL of MB (0.05 mM) was examined prior to adding hydrogel and 0.5 mL of NaBH_4_. To monitor the reaction progress, a UV-Vis spectrophotometer was used. MB solutions were measured at 665 nm and the UV-vis cuvette shows where the catalytic reduction took hold. Numerous experimental parameters were examined and optimized, including initial MB concentration (0.01, 0.05 mM), dose amount (5, 10 and 15 mg), and reducing agent NaBH_4_ concentration (0.05, 0.1 and 0.2 M). All chemicals’ percent conversion (% R) was determined using Equation (1):

(1) $$\%\;Conversion=\frac{C_{0}-C_{t}}{C_{0}}*100$$

To quantify the rate of these reactions, the UV–visible spectral data were treated according to Equation (2):

(2) $$\ln\frac{C_{t}}{C_{0}}=-Kt$$

where *C*_0_ and *C_t_* are the absorbance values at 665 nm noted from the UV–visible spectra of MB dye solution before and after addition of the reductant and catalyst, and *C_t_* and *C*_0_ represent the concentration, respectively.

## 3. Result and Discussion

### 3.1. Characterization

#### 3.1.1. Field Emission Scanning Electron Microscope (FE-SEM)

**Figure 2:**
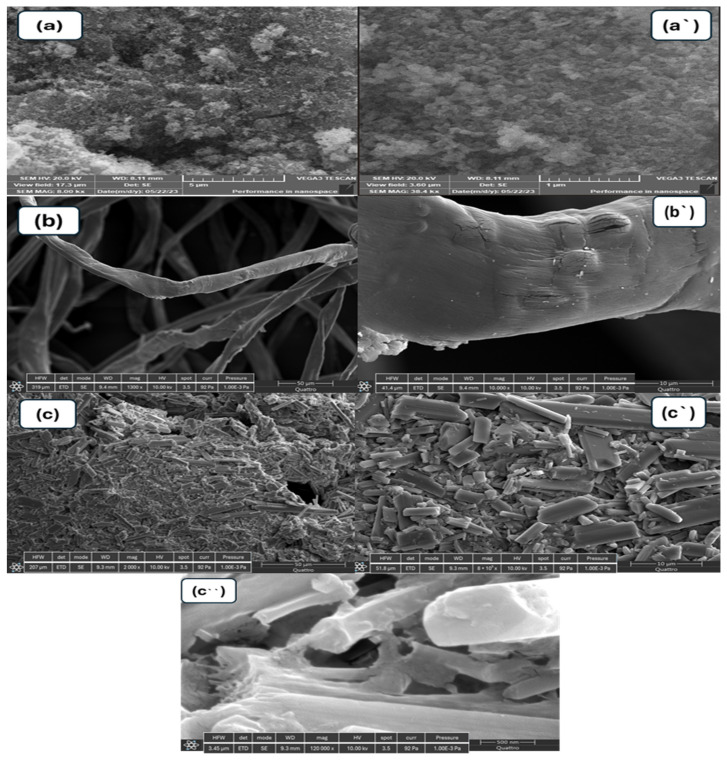
SEM images (**a**,**a`**) CuO−NiO, (**b**,**b`**)GG hydrogel, and (**c**,**c`**,**c``**) GG−CuO−NiO hydrogel.

**Figure 3:**
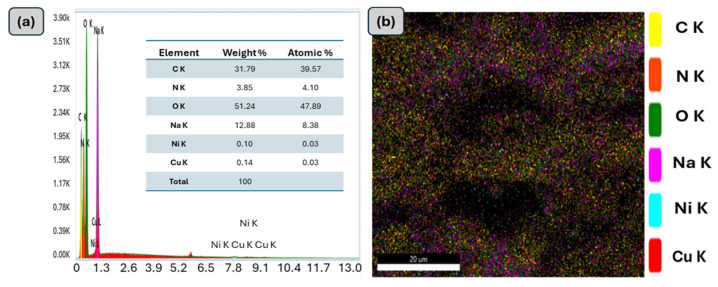
(**a**) EDS and (**b**) EDS mapping of GG−CuO−NiO hydrogel.

The surface morphology of the prepared materials, CuO-NiO nanocomposite, and GG-CuO-NiO hydrogel were analyzed using SEM, as shown in Figure 2a–c. The high-magnification SEM images are shown on the left side, while the corresponding low-magnification images are displayed on the right. The images in Figure 2a,a` show the particles of the CuO-NiO nanocomposite, which predominantly exhibit a globular morphology [32]. The image of the GG-CuO-NiO hydrogel was compared to the pure GG hydrogel in Figure 2b,b` revealing a smoother surface and less roughness than the GG surface. The GG-CuO-NiO hydrogel chains became more overlapping, giving the hydrogel a strong structure. Moreover, the CuO-NiO nanocomposite was planted well on the GG hydrogel, as shown in Figure 2c,c`,c``. The surface mapping of the GG-CuO-NiO nanocomposite is depicted in Figure 3a,b, which also shows the elements that are present, including carbon (C), nitrogen (N), sodium (Na), copper (Cu), nickel (Ni), and oxygen (O).

#### 3.1.2. X-Ray Diffraction XRD

**Figure 4:**
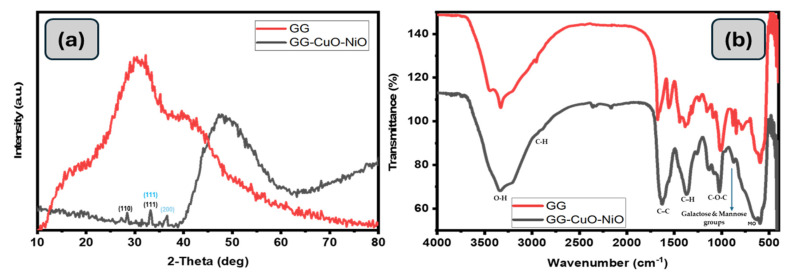
(**a**) XRD pattern of the nanocomposites, (**b**) FT-IR images for prepared materials GG and GG−CuO−NiO hydrogel.

The crystal structures and phase purities of GG and GG-CuO-NiO hydrogel were examined by X-ray diffraction, and the XRD in image Figure 4a for GG shows a broad reflection at 20.6° and a hump at 40.0°, which corresponds to the amorphous nature of the guar gum. The characteristic diffraction peaks of CuO and NiO observed in the hydrogel nanocomposite match those previously reported for the pure CuO-NiO nanocomposite [32]. Meanwhile, the XRD pattern of the GG-CuO-NiO hydrogel shows a significant shift in the broad GG peak from 20.6° to 40.0°, indicating strong interactions between the polymer matrix and the incorporated metal oxides. Furthermore, low-intensity diffraction peaks at 2θ = 36.7° (111) and 43.3° (200) correspond to NiO in the cubic phase, JCPDS card 47-1049 [33]. In contrast, peaks at 2θ = 32.2° (110), 36.6° (111), and 38.6° (202) confirm the presence of CuO in its monoclinic structure, JCPDS card 48-1548 [33,34,35,36,37]. The predominant amorphous character of GG is responsible for the suppression of these peaks, which lessens their intensity. The Debye–Scherrer equation was used to determine the crystallite size of the GG-CuO-NiO hydrogel:

D = Kλ/βcos θ

where D is the crystallite size (nm), K is the Scherrer constant (0.94), λ is the X-ray wavelength (1.54 Å), β is the full-width at half-maximum (FWHM) in radians, and θ is the Bragg angle in degrees [38]. The average crystallite size of the GG-CuO-NiO hydrogel was calculated to be 17 nm.

#### 3.1.3. Fourier-Transform InfraRed (FT-IR) Spectroscopy

Figure 4b displays the FT-IR spectra of GG and the GG-CuO-NiO hydrogel. A few of the distinctive bands of GG-CuO-NiO resemble GG, albeit with some shifting. The O-H stretching vibration band was detected at 3334 cm^−1^ for GG; however, it was moved to 3349 cm^−1^. The interaction between the CuO-NiO group and the GG-CuO-NiO hydrogel is responsible for the shift in the O-H group. For GG and GG-CuO-NiO, the band linked to the C-H stretching vibration is located at 2992 cm^−1^ and 2897 cm^−1^, respectively, in a comparable wavenumber. For GG, the band that represents the C–C ring stretching vibration was detected at 1674 cm^−1^; for GG-CuO-NiO, it was slightly displaced to 1651 cm^−1^. For GG and GG-CuO-NiO, the C–H stretching band was detected at 1566 cm^−1^ and 1549 cm^−1^, respectively. The C-O-C stretching vibration band in GG and GG-CuO-NiO was found at 1019 cm^−1^ and 1025 cm^−1^, respectively. At a similar wavenumber for GG-CuO-NiO, the bands indicating galactose and mannose groups were detected at 846 cm^−1^ and 779 cm^−1^. These bands were also detected at 858 cm^−1^ and 810 cm^−1^ [37].

### 3.2. Catalytic Reduction Study

#### Discoloring Study of Dyes

**Figure 5:**
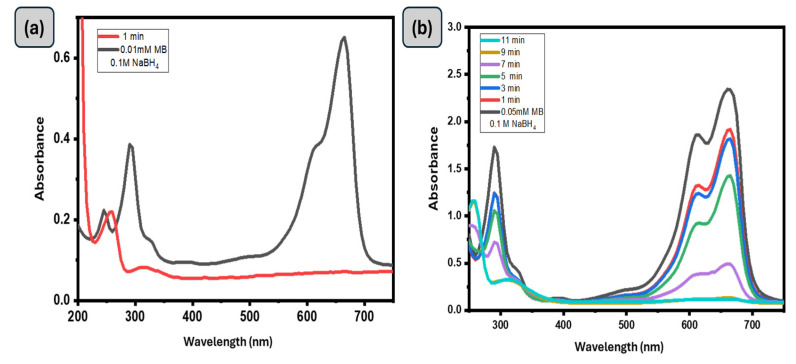

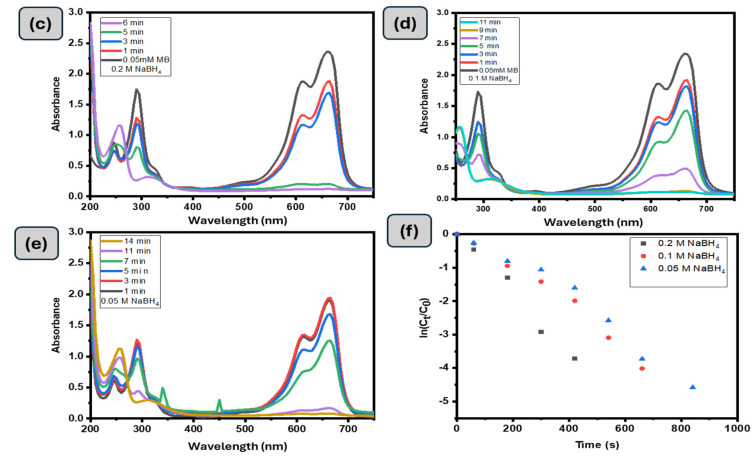
(**a**,**b**): UV-Vis spectra of (0.01 mM and 0.05 mM) MB using 0.1 g of GG−CuO−NiO hydrogel in the presence of 0.1 M NaBH_4_, (**c**–**e**) UV-Vis spectra of (0.05 mM) MB using 0.1 g of GG−CuO−NiO hydrogel in the presence of (0.05 M, 0.1 M and 0.2 M) NaBH_4_, (**f**) kinetics of (0.05 mM) MB using 0.1 g of GG−CuO−NiO hydrogel in the presence of (0.05 M, 0.1 M and 0.2 M) NaBH_4_.

The catalytic activity of the GG-CuO-NiO hydrogel, in breaking down various types of dyes, like Methylene Blue (MB), which is a cationic dye, was chosen as a model compound for this study. An aqueous solution of MB that exhibits a shoulder at 614 nm and an absorbance peak at 664 nm was employed. It was previously found that the n → π* transition of MB is associated with the primary absorbance peak at 664 nm [39,40]. Plotting the absorbance peak at 664 and 614 nm against time allows one to assess the rate of the MB reduction reaction. A range of MB concentrations, NaBH_4_ concentrations, and GG-CuO-NiO hydrogel amount were used in the reduction tests to maximize the ideal circumstances for high catalytic activity. Initially, the catalytic reduction of MB dye was studied in the absence of NaBH_4_, and no noticeable reaction was observed even after 1 h. Subsequently, the reaction was tested in the absence of the GG-CuO-NiO hydrogel, and similarly, no significant reduction occurred, thus demonstrating that both NaBH_4_ and the catalyst are necessary for the reduction process. Figure 5 illustrates the significant degradation of MB dye with NaBH_4_ in the presence of the GG-CuO-NiO hydrogel as a catalyst. Thus, the presence of 0.5 mL of (0.1M) NaBH_4_ and 0.1 g of GG-CuO-NiO hydrogel was shown to achieve excellent catalytic degradation. The addition of GG-CuO-NiO is shown to accelerate the reduction process, as evidenced by a significant drop in the absorption band. The full reduction of MB was achieved in 1 min when GG-CuO-NiO was present, according to the plot of absorbance intensity against wavelength at regular intervals of time (1 min), as shown in Figure 5. The reduction is a pseudo first-order reaction, as seen by the linear relationship between ln(A_0_/A_t_) and reaction time. The efficiency of the reduction process with the GG-CuO-NiO hydrogel is highly dependent on the concentrations of the reducing agent and the pollutant, as they directly impact the reaction kinetics and overall degradation effectiveness. Consequently, 0.1 g of GG-CuO-NiO hydrogel was used for (MB 0.05, and 0.01) mM in the presence of (0.1 M) NaBH_4_. As seen in Figure 5, the reduction percentage varied from 80% to 99% for all values that were investigated.

Furthermore, the concentration of the reducing agent (NaBH_4_) can have a significant influence on the catalytic reduction of MB dye. For this effect, various NaBH_4_ concentrations (0.05, 0.1, and 0.2 M) and the same amount of GG-CuO-NiO hydrogel were explored for their ability to reduce the 0.05 mM MB dye molecules. The catalytic reduction rate constants of MB were found to be 1.29 × 10^−2^, 6.12 × 10^−3,^ and 6.31 × 10^−3^ (s^−1^). Higher NaBH_4_ concentrations resulted in a faster rate of reduction in a shorter period of time than lower concentrations, as seen in Figure 5c. The comparison of catalytic MB reduction by GG-CuO-NiO with other reported NPs-based catalysts is summarized in Table 1.

**Figure 6:**
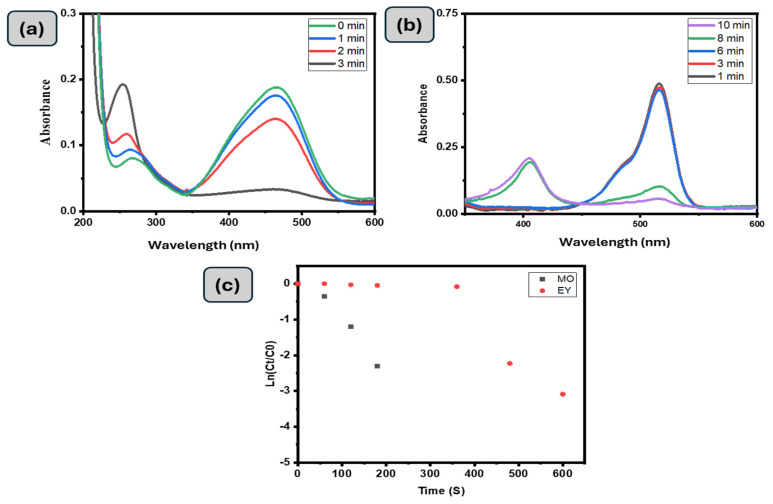
UV-Vis spectra of the (**a**) (0.01 mM) MO and (**b**) (0.01 mM) EY degradation with 0.1 g of GG−CuO−NiO hydrogel in the presence of 0.1 M NaBH_4_, (**c**) kinetic MO, and EY with 0.1 g of GG−CuO−NiO hydrogel in the presence of 0.1 M NaBH_4_.

A GG-CuO-NiO hydrogel was employed to investigate also toward the catalytic reduction of Methyl Orange (MO). The UV-visible spectrum of MO treated with GG-CuO-NiO hydrogel is displayed in Figure 6a, along with a visual representation of it over time. The greatest wavelength of visible absorption for the chromophore group was 464 nm, with absorbance at 270 nm caused by π to π* transitions associated with rings. Figure 6a shows that the absorbance for these bands at 464 and 270 nm progressively decreased over time, indicating that the GG-CuO-NiO hydrogel either adsorbed or disrupted the conjugated structure of MO. At the same time, a new absorption peak at 248 nm was developed in accordance with the sulfonic acid solution UV spectra [34,39]. This peak gradually increased in strength, suggesting that sulfonic acid was one of the products and that reduction was a key factor in the decolorization process. In around 180 s, the quickly fading intensity band at 468 nm eliminated more than 90% of MO. It was determined that the rate constant for the pseudo first-order process was 1.29 × 10^−2^ s^−1^.

The catalytic activity of Eosin Yellow (EY) by the GG-CuO-NiO hydrogel in the presence of NaBH_4_ was explored (Figure 6b). There was an absorption peak of EY at 510 nm. However, once 0.1 g of GG-CuO-NiO hydrogel was added to the mixture of EY and NaBH_4_, the abortion peak of EY gradually decreased. Throughout the reduction process, the color of the EY solution gradually disappeared. As seen in Figure 6b, within 10 min, 80% of the EY peak at 510 nm was eliminated. It was found that the first order reaction was determined to be 6.39 × 10^−3^ s^−1^.

**Figure 7:**
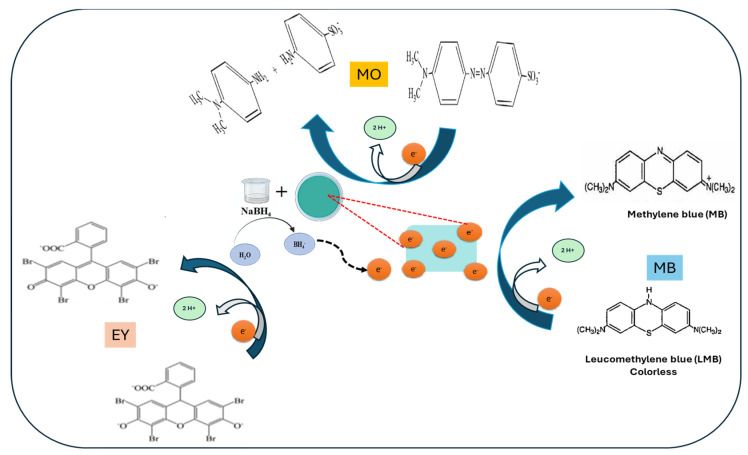
Mechanism for the catalytic reduction of selected dyes; (MB, MO and EY) by GG-CuO-NiO hydrogel in the presence of NaBH_4_.

The possible mechanism for the catalytic reduction of the select dyes, Methylene Blue (MB), Methyl Orange (MO), and Eosin Yellow (EY), primarily involves nanocomposite catalyst-mediated electron transfer. Initially, the ions produced from the NaBH_4_ dissociate in aqueous solution. NaBH_4_ functions as an electron donor to produce reactive hydrogen species (BH_4_⁻ or H⁻), while GG-CuO-NiO enables fast electron transfer to dye molecules to speed up their reduction. In the case of MB, reversible π-π stacking interactions between the π-conjugated MB molecules and the GG-CuO-NiO hydrogel on the nanocatalyst allow them to attach to the GG-CuO-NiO hydrogel surface. The BH_4_⁻ ions provide the MB molecules electrons via the GG-CuO-NiO-BH_4_ species once both MB molecules and BH₄⁻ ions have been adsorbed on the surface. This electron transfer makes it easier for MB to be reduced into its colorless Leucomethylene Blue (LMB) form, effectively completing the reduction process. In addition, the azo (-N = N-) bond in MO undergoes cleavage to produce colorless aromatic amines. The disruption of π-conjugation in EY results in the loss of color. The catalytic properties of GG-CuO-NiO boost reaction rates, which leads to efficient and fast dye destruction, as shown in Figure 7.

### 3.3. Catalytic Stability

**Figure 8:**
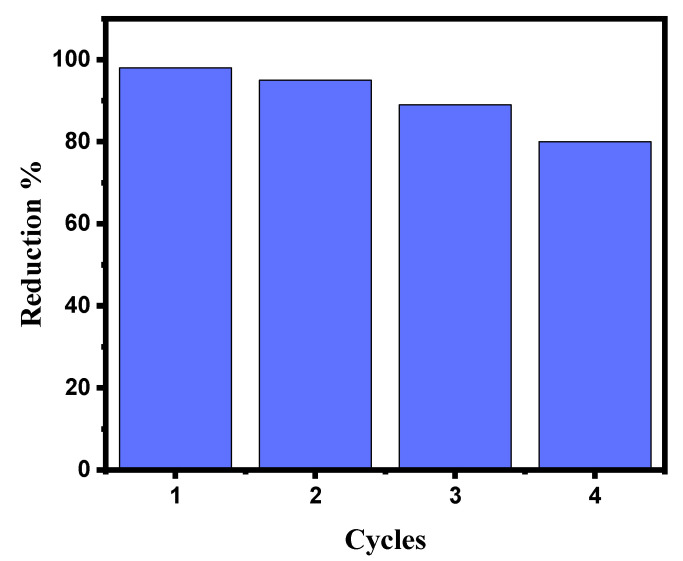
Recyclability of GG-CuO-NiO hydrogel toward the reduction of MB in the presence of NaBH_4_.

Catalytic stability is just as important as catalyst activity when assessing the catalyst’s effectiveness and its uses. To reduce MB, the stability of the GG-CuO-NiO hydrogel was examined. According to the results, the GG-CuO-NiO hydrogel could be recycled up to three times in the same way as the experimental section for the catalytic reduction of (0.01 mM) MB in the presence of (0.1 M) NaBH_4_. Before the GG-CuO-NiO hydrogel was assessed for the third cycle, it was cleaned three times with distilled water after each use. As seen in Figure 8, the catalytic reduction for the first use of (0.01 mM) MB took 1 min, while the subsequent uses took 2.5, 4, and 6 min the second, third, and fourth times, respectively. This suggests that the catalyst was still actively and successfully decreasing MB. As a result, up to fourth cycle of activation, the GG-CuO-NiO hydrogel remained feasible.

### 3.4. Catalytic Efficiency of GG-CuO-NiO Hydrogel in Real Samples

The effectiveness of the GG-CuO-NiO hydrogel was assessed under ideal conditions using three real sample types, orange juice, tap water, and sea water, from a Jazan market and the Jazan Laboratory in Saudi Arabia. To create the actual samples, each type of sample was diluted separately using 1 mL in 100 mL of deionized water. Following the transfer of 2.0 mL of the actual sample solution to UV cuvettes, 0.5 mL of MB (0.01 mM) and 0.5 mL of (0.1 M) NaBH_4_ were added. Finally, the mixture was supplemented with 0.1 g of the GG-CuO-NiO hydrogel. Using a UV-vis spectrophotometer, the catalytic reduction of the hydrogel was seen. Table 2 provides a summary of all the collected data. According to the findings, the GG-CuO-NiO hydrogel was reliable and reasonable for reducing real samples, including orange juice, tap water, and sea water.

## 4. Conclusions

This study successfully developed a guar gum-based GG-CuO-NiO hydrogel using formaldehyde as a crosslinking agent, demonstrating remarkable catalytic efficiency for dye reduction in the presence of NaBH₄. Structural and morphological analyses using SEM, EDX, XRD, and FT-IR confirmed the hydrogel’s composition. The hydrogel exhibited rapid and efficient dye degradation, achieving a 95% removal of MB within one minute, while MO and EY showed 90% and 80% removal in four and ten minutes, respectively. The catalytic activity was further optimized by adjusting the dye concentration, reducing agent dosage, and hydrogel quantity. Reusability studies show that the hydrogel remained stable over four cycles, with a minimal loss of efficiency. Moreover, its performance in real sample applications demonstrated excellent reduction capabilities, highlighting its potential as a sustainable and effective catalyst for wastewater treatment.

## Figures and Tables

**Table 1:** Comparison the catalytic reduction toward MB using GG-CuO-NiO hydrogel in the presence of NaBH_4_ with other reported catalysts.

Pollutants	Catalyst	Time (Sec)	Rate Constant S^−1^	Ref.
MB	CuO–SDS ^1^	600	5.75 × 10^−3^	[40]
MB	CuO@CS ^2^	600	5.75 × 10^−3^	[41]
MB	Ag/PSZN-5 ^3^	240	17.29 × 10^−3^	[42]
MB	Fe_3_O_4_/ZIF-67@ZnO	540	2.23 × 10^−3^	[38]
MB	CuO-NiO	180	1.195 × 10^−2^	[43]
MB	GG-CuO-NiO Hydrogel	60	5.67 × 10^−2^	This study

^1^ CuO nanoparticles—sodium dodecyl sulfate (SDS). ^2^ CuO nanoparticles supported on cellulose kitchen wipe sponge. ^3^ Pure silicon zeolite nanoparticle (PSZN).

**Table 2:** Application of the catalytic reduction of MB in real samples utilizing GG-CuO-NiO.

Real Samples	Reduction Time (s)	Reduction %
Juice	85	87
Tap water	93	95
Sea water	79	90

## Data Availability

The data presented in this study are available on request from the corresponding author.
